# Temporal Changes of Cardiac Structure, Function, and Mechanics During Sub-acute Cervical and Thoracolumbar Spinal Cord Injury in Humans: A Case-Series

**DOI:** 10.3389/fcvm.2022.881741

**Published:** 2022-06-15

**Authors:** Shane J. T. Balthazaar, Tom E. Nightingale, Katharine D. Currie, Christopher R. West, Teresa S. M. Tsang, Matthias Walter, Andrei V. Krassioukov

**Affiliations:** ^1^International Collaboration on Repair Discoveries, University of British Columbia, Vancouver, BC, Canada; ^2^Experimental Medicine Program, Faculty of Medicine, University of British Columbia (UBC), Vancouver, BC, Canada; ^3^School of Sport, Exercise, and Rehabilitation Sciences, University of Birmingham, Birmingham, United Kingdom; ^4^Centre for Trauma Science Research, University of Birmingham, Birmingham, United Kingdom; ^5^Department of Kinesiology, Michigan State University, East Lansing, MI, United States; ^6^Department of Cellular and Physiological Sciences, Faculty of Medicine, UBC, Vancouver, BC, Canada; ^7^Department of Cardiology, Vancouver General and UBC Hospitals, Vancouver Coastal Health, Vancouver, BC, Canada; ^8^Department of Urology, University Hospital Basel, University of Basel, Basel, Switzerland; ^9^Division of Physical Medicine and Rehabilitation, Faculty of Medicine, UBC, Vancouver, BC, Canada; ^10^GF Strong Rehabilitation Centre, Vancouver Coastal Health, Vancouver, BC, Canada

**Keywords:** spinal cord injuries, echocardiography, ventricular function, time course, autonomic nervous system

## Abstract

Individuals with cervical spinal cord injury (SCI) experience deleterious changes in cardiac structure and function. However, knowledge on when cardiac alterations occur and whether this is dependent upon neurological level of injury remains to be determined. Transthoracic echocardiography was used to assess left ventricular structure, function, and mechanics in 10 male individuals (median age 34 years, lower and upper quartiles 32–50) with cervical (*n* = 5, c-SCI) or thoracolumbar (*n* = 5, tl-SCI) motor-complete SCI at 3- and 6-months post-injury. Compared to the 3-month assessment, individuals with c-SCI displayed structural, functional, and mechanical changes during the 6-month assessment, including significant reductions in end diastolic volume [121 mL (104–139) vs. 101 mL (99–133), *P* = 0.043], stroke volume [75 mL (61–85) vs. 60 mL (58–80), *P* = 0.042], myocardial contractile velocity (S') [0.11 m/s (0.10–0.13) vs. 0.09 m/s (0.08–0.10), *P* = 0.043], and peak diastolic longitudinal strain rate [1.29°/s (1.23–1.34) vs. 1.07°/s (0.95–1.15), *P* = 0.043], and increased early diastolic filling over early myocardial relaxation velocity (E/E') ratio [5.64 (4.71–7.72) vs. 7.48 (6.42–8.42), *P* = 0.043]. These indices did not significantly change in individuals with tl-SCI between time points. Ejection fraction was different between individuals with c-SCI and tl-SCI at 3 [61% (57–63) vs. 54% (52–55), *P* < 0.01] and 6 months [58% (57–62) vs. 55% (52–56), *P* < 0.01], though values were considered normal. These results demonstrate that individuals with c-SCI exhibit significant reductions in cardiac function from 3 to 6 months post-injury, whereas individuals with tl-SCI do not, suggesting the need for early rehabilitation to minimize cardiac consequences in this specific population.

## Introduction

Left ventricular (LV) atrophy and decreased volumes are well documented in individuals with chronic spinal cord injury (SCI) (1). Decreased functional outcomes can result from impaired supraspinal sympathetic control over the cardiovascular system, such that cardiac responses to stress or exercise are diminished in individuals with high-level SCI (i.e., injury above the 6th thoracic spinal cord level) compared with non-injured individuals (2). Compounded with the higher prevalence of physical inactivity in the SCI population (3), it is perhaps unsurprising that cardiovascular disease (CVD) is the leading cause of morbidity and mortality in this population (4). These aforementioned factors have more substantial effects for individuals with cervical SCI compared to thoracolumbar SCI due to the combination of impaired sympathetic control to the heart (5), lack of skeletal muscle pump (i.e., decrease in venous return) (6), and greater functional impairments limiting physical activity (7). These factors predispose individuals with cervical SCI to daily cardiovascular disturbances, consequentially resulting in outcomes such as autonomic dysreflexia, orthostatic hypotension, and pooling of blood below the level of injury (8). Ultimately, previous literature suggests that these events can lead to detrimental cardiac consequences (9–11).

Similar to SCI, microgravity exposure during spaceflight has been associated with an overall physiological decline, providing a potential model to better understand the alterations in these specific populations over time (12). While the time course of changes in the autonomic nervous system following spaceflight and SCI have been described (12), temporal echocardiographic changes shortly after SCI have not been followed longitudinally. This is surprising as other cardiac changes, such as an increased prevalence of arrhythmias, occur in the months following SCI (13). Furthermore, previous research has demonstrated reduced echocardiographic indices of LV systolic function in individuals with cervical SCI at the chronic stage of injury (>1 year) compared to non-injured individuals (1), potentially due to the disrupted sympathetic input to the heart originating from T1 to T5 (14). Recently, a cross-sectional study revealed associations between increased time since injury and reduced LV size and function; these alterations have been linked to consequences such as congestive heart failure and increased mortality (15). Ultimately, the cardiac consequences (1) of decreased cardiac reserve has important clinical implications as it can limit the ability of an individual to perform regular activities of daily living (16). Furthermore, characterizing the time frame of structural and functional cardiac alterations may provide further insight into the increased risk of CVD in those with SCI and aid in the timing of cardiac rehabilitation interventions.

No study to date has examined the longitudinal changes in cardiac function post-SCI in the sub-acute setting (i.e., within the first 6 months), stratifying for neurological level of injury (NLI). Thus, the aim of this present longitudinal case series was to investigate changes in LV structure, function, and mechanics, in individuals with cervical SCI and thoracolumbar SCI at 3- and 6-months post-injury using transthoracic echocardiography (TTE) (17).

## Materials and Methods

### Participants

Clinical protocols were approved by the University of British Columbia Clinical Research Ethics Board (H13-03072) and conducted in accordance with the second Helsinki Declaration. The participants provided written informed consent prior to data collection. The NLI and the severity of SCI were classified according to the International Standards for Neurological Classification of SCI (ISNCSCI) by a trained physician [i.e., providing an American Spinal Injury Association Impairment Scale (AIS) grade] (18). Participants were asked to report the frequency and duration of moderate (some physical effort) and heavy (maximum physical effort) intensity leisure time physical activity (LTPA) over the preceding 7 days using the validated LTPA questionnaire for individuals with SCI (LTPAQ-SCI) (19). These data provide an indication of the volume of weekly LTPA performed by participants. The validity of the LTPAQ-SCI is comparable to questionnaires used in the general population (19). Exclusion criteria for all participants included any history of CVD and any language or cognitive barrier that prevented the participant from following English instructions.

### Echocardiography

TTE was performed on a commercially available ultrasound (Vivid 7/i; GE Medical, Horton, Norway) and stored for offline analysis using specialized computer software (EchoPAC; GE Healthcare, Horton, Norway) according to the recommendations of the American Society for Echocardiography (ASE) (20), by a single analyzer, blinded to time point and group.

The average of three cardiac cycles was used to determine LV structure and functional indices. Measures of LV structure at end-diastole and end-systole were reported from the parasternal long-axis views, and used to derive relative wall thickness (21). Volumetric measurements and systolic functions were derived from the apical four- and two-chamber views using the modified Simpson's biplane method. Cardiac output (Q) was calculated as the product of stroke volume (SV) and heart rate (HR) and systolic myocardial velocity (S') was derived from pulsed-wave Doppler. LV diastolic indices were calculated using early septal relaxation velocity (E'), early (E) and late (A) transmitral flow, and early-to-late transmitral filling velocity (E/A) ratio derived from pulsed-wave Doppler. The E/E' ratio was calculated to estimate LV filling pressure. Deceleration time and isovolumetric relaxation time were also determined on the spectral Doppler trace. LV mass index was calculated with the Devereux method and indexed to body surface area using the DuBois method (22, 23).

Indices of LV mechanics were derived from apical four-chamber and parasternal short-axis images at the level of the mitral valve (basal), papillary muscle (mid), and apex (apical). Images were analyzed using 2D speckle-tracking software in accordance with recommended guidelines (24). Raw speckle-tracking traces were imported into customized post-processing software (2D Strain Analysis Tool, Stuttgart, Germany), and data was interpolated into 600 points in systole and 600 points in diastole using a cubic spline algorithm. Peak strain and strain rate in systole and diastole were determined for each parasternal short-axis view (radial, circumferential) and the apical four-chamber view (longitudinal). Basal and apical peak rotation and rotation rate in systole and diastole were determined. Twist was determined as the maximum value obtained when subtracting the frame-by-frame basal rotation from the frame-by-frame apical rotation. Torsion, a measure of twist normalized to LV chamber size, was calculated by dividing peak twist by the LV end-diastolic length.

### Statistical Analysis

Data are presented as raw values and percentages. Statistical analyses were performed using Statistical Package for Social Science software (SPSS Version 27, IBM, Chicago, IL, USA) with statistical significance set at *P* ≤ 0.05. Wilcoxon signed rank test were used to analyze outcomes between time points within groups. Group differences were analyzed using the Mann Whitney U-test. Results are presented as median with upper and lower quartiles (i.e., 25–75%). Graphical representations were made in Prism (Version 9.1.1, GraphPad Software, San Diego, CA), SPSS, and Adobe Illustrator (Version 25.2.3, Adobe Inc., San Jose, CA).

## Results

### Participant Characteristics

A total of 10 male participants (median age 34 years, 32–50) with motor-complete (AIS A/B) cervical SCI (*n* = 5) or thoracolumbar SCI (*n* = 5) were included. TTE was conducted 79 days (65–108) and 204 (191–218) post-injury. Demographics are highlighted in Table 1, with no significant differences observed between groups.

**Table 1:** Demographics, injury characteristics, and perceived physical activity level of participants.

**Participant**	**NLI**	**AIS**	**Sex**	**Age** **(years)**	**Height** **(cm)**	**Weight** **(kg)**	**At 3-months**		**At 6-months**	
							**MH-LTPA (min/week)**	**UEMS** **(max. score 50)**	**MH-LTPA (min/week)**	**UEMS** **(max. score 50)**
1	C6	A	M	28	188	82	80	24	360	24
2	C5	A	M	41	180	102	120	12	0	18
3	C5	A	M	35	183	80	840	24	360	24
4	C4	B	M	59	180	98	0	19	0	40
5	C5	A	M	32	186	78	39	7	160	11
Median (LUQ)	–	–	–	35 (30–50)	183 (180–187)	82 (79–100)	80 (20–480)	19 (10–24)	160 (0–360)	24 (15–32)
6	T9	A	M	23	183	88	180	50	360	50
7	T7	A	M	50	170	72	450	50	0	50
8	T9	A	M	33	190	136	0	50	0	50
9	L1	A	M	59	179	56	0	50	0	50
10	L1	B	M	33	180	72	0	50	180	50
Median (LUQ)	–	–	–	33 (28–55)	180 (175–187)	72 (67–112)	0 (0–315)	50	0 (0–270)	50

*The cervical and thoracolumbar groups were male and not significantly different with respect to age (P = 1.00), height (P = 0.40), weight (P = 0.35), and at each measurement timepoint*.

*The severity (AIS grade) and NLI as well as UEMS were assessed in accordance with the International Standards for Neurological Classification of spinal cord injury (ISNCSCI)*.

*AIS, American Spinal Injury Association Impairment Scale; LUQ, lower and upper quartiles; MH-LTPA, moderate-to-heavy leisure time physical activity; NLI, neurological level of injury; UEMS, upper motor extremity score*.

### Changes in Cardiac Structure, Function, and Mechanics

**Figure 1:**
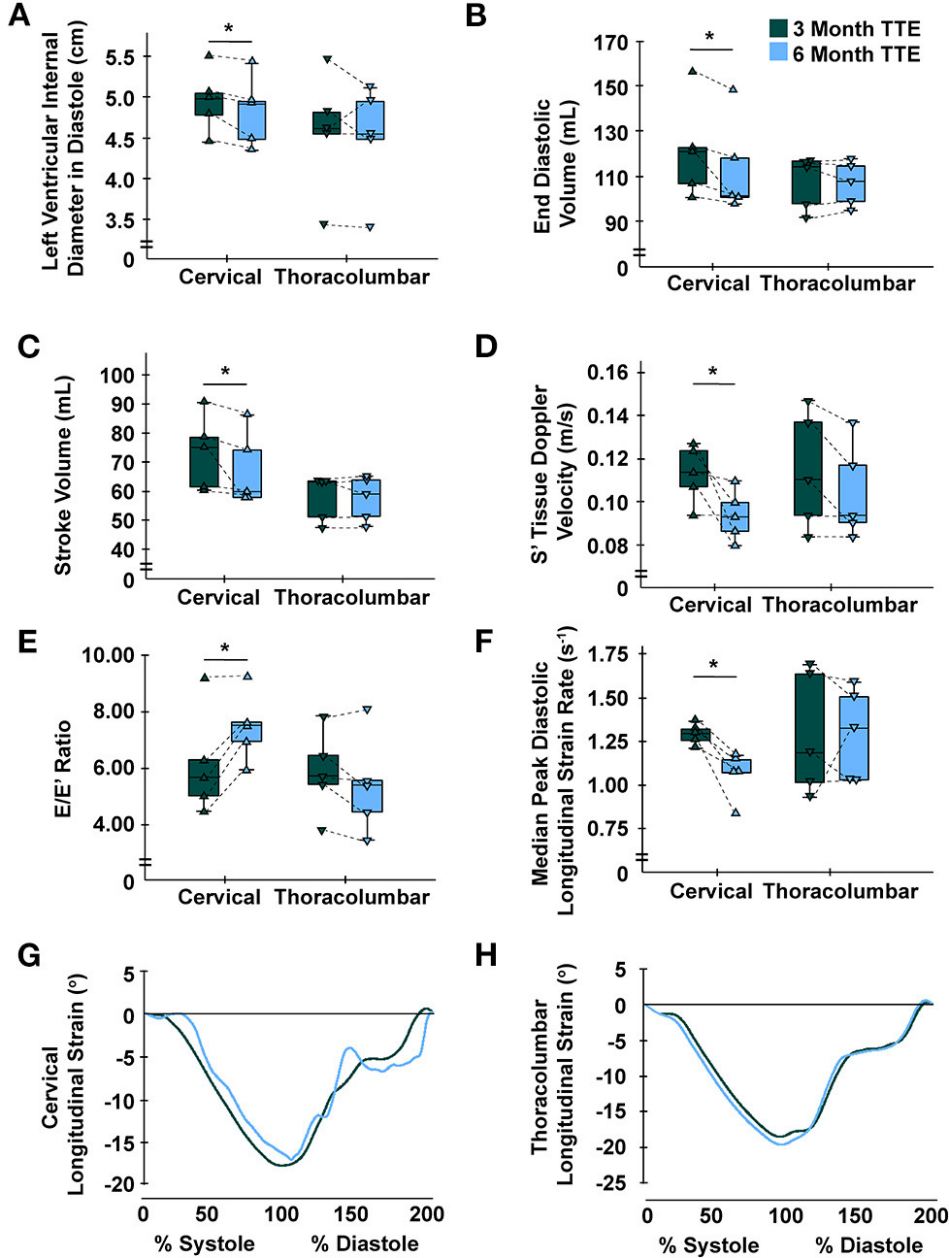
Time-course alterations following cervical spinal cord injury at 6-months for left ventricular indices. **(A)** Left ventricular internal diameter in diastole was significantly reduced in the cervical group at 6- vs. 3-months, yet not in the thoracolumbar group. **(B)** End diastolic volume was significantly reduced in the cervical group at 6- vs. 3-months, yet not in the thoracolumbar group. **(C)** Stroke volume was significantly reduced in the cervical group at 6- vs. 3-months, yet not in the thoracolumbar group. **(D)** Myocardial contractile velocity (S') was significantly reduced in the cervical group at 6- vs. 3-months, yet not in the thoracolumbar group (25). **(E)** Early diastolic filling over early myocardial relaxation velocity (E/E') ratio was significantly increased in the cervical group at 6- vs. 3-months, yet not in the thoracolumbar group. **(F)** Median peak diastolic longitudinal strain rate was significantly lower at 6- vs. 3-months in the cervical group, yet not in the thoracolumbar group. **(G,H)** Global longitudinal strain standardized to cardiac cycle length for all participants at the 3- and 6-month time points for the cervical **(G)** and thoracolumbar **(H)** SCI groups; no differences were observed within groups. C, cervical; L, lumbar; S, sacral; T, thoracic; TTE, transthoracic echocardiography. * Cervical 6-months different from cervical 3-months (*P* < 0.05). Data are displayed individually (i.e., each triangle represents one individual) and grouped (i.e., box-and-whisker plot).

At 3- vs. 6-months post-injury, TTE revealed a significant decrease in LV internal diameter in diastole (LVIDd) [5.0 cm (4.6–5.3) vs. 4.9 (4.4–5.2), *P* = 0.043], end-diastolic volume (EDV) [120.83 mL (103.58–139.42) vs. 101.33 (99.17–133.18), *P* = 0.043], end-systolic volume (ESV) [45.67 mL (41.42–55.83) vs. 43.33 (40.17–52.75), *P* = 0.043], SV [75.17 mL (61.08–84.67) vs. 60.00 (58.00–80.33), *P* = 0.042], S' [0.11 m/s (0.10–0.13) vs. 0.09 (0.08–0.10), *P* = 0.043], and increased E/E' ratio [5.64 (4.71–7.72) vs. 7.48 (6.42–8.42), *P* = 0.043] for individuals with cervical SCI. For LV diastolic mechanics, longitudinal strain rate was lower in the cervical group at three compared to 6 months [1.29 degrees/s (1.23–1.34) vs. 1.07 (0.95–1.15), *P* = 0.043] (Figure 1). These indices did not significantly change in individuals with thoracolumbar SCI between time points. Further indices for LV dimensions, systolic function, and diastolic function are highlighted in Table 2.

**Table 2:** Echocardiographic indices for LV structure and function between cervical and thoracolumbar SCI groups and within 3- and 6-month time points.

	**GROUP**	**Time since injury** **3 months**	**Time since injury** **6 months**
**Heart rate measures**			
HR (bpm)	All SCI	72 (58–79)	66 (63–78)
	Cervical	64 (51–74)	64 (50–74)
	Thoracolumbar	77 (65–91)	76 (66–82)
**LV volumetric and systolic measures**			
EDV (mL)	All SCI	115 (100–121)	**105 (99**–**118)**^†^
	Cervical	121 (104–139)	**101 (99**–**133)**^†^
	Thoracolumbar	114 (95–117)	108 (97–116)
ESV (mL)	All SCI	46 (44–53)	47 (43–51)
	Cervical	46 (41–56)	**43 (40–53)** ^†^
	Thoracolumbar	50 (45–53)	49 (47–51)
SV (mL)	All SCI	64 (58–76)	60 (56–67)
	Cervical	75 (61–84)	**60 (58–80)** ^†^
	Thoracolumbar	63 (50–64)	59 (50–65)
Q (L/min)	All SCI	4.5 (3.6 t- 5.9)	4.4 (3.4–4.8)
	Cervical	5.0 (3.1–6.1)	4.8 (3.0–5.2)
	Thoracolumbar	4.0 (3.7–5.6)	4.3 (3.7–4.7)
EF (%)	All SCI	56 (54–62)	57 (54–59)
	Cervical	61 (57–63)	58 (57–62)
	Thoracolumbar	54 (52–55)**	55 (52–56)**
S' septal (m/s)	All SCI	0.11 (0.09–0.13)	**0.09 (0.08–0.11)** ^†^
	Cervical	0.11 (0.10–0.13)	**0.09 (0.08–0.10)** ^†^
	Thoracolumbar	0.11 (0.09–0.14)	0.09 (0.09–0.13)
**LV diastolic measures**			
E (cm/s)	All SCI	73 (59–80)	**59 (53–70)** ^†^
	Cervical	75 (66–95)	58 (50–74)
	Thoracolumbar	62 (55–73)	60 (52–69)
Deceleration time (ms)	All SCI	190 (141–228)	219 (185–269)
	Cervical	214 (130–238)	217 (208–248)
	Thoracolumbar	167 (146–243)	260 (141–304)
A (cm/s)	All SCI	51 (42–57)	47 (43–63)
	Cervical	46 (40–54)	45 (39–51)
	Thoracolumbar	53 (38–68)	62 (45–71)
E/A	All SCI	1.47 (1.23–2.08)	**1.29 (0.99–1.48)** ^†^
	Cervical	1.58 (1.40–2.15)	1.31 (1.16–1.61)
	Thoracolumbar	1.37 (0.83–1.91)	1.13 (0.73–1.45)
IVRT (ms)	All SCI	58 (55–81)	64 (57–73)
	Cervical	76 (54–100)	62 (59–77)
	Thoracolumbar	57 (50–61)	66 (56–70)
E' septal (m/s)	All SCI	0.14 (0.09–0.16)	0.10 (0.07–0.13)
	Cervical	0.16 (0.11–0.17)	0.09 (0.07–0.10)
	Thoracolumbar	0.11 (0.09–0.14)	0.13 (0.09–0.15)
E/E'	All SCI	5.67 (4.89–6.77)	6.42 (5.13–7.72)
	Cervical	5.64 (4.71–7.72)	**7.48 (6.42–8.42)** ^†^
	Thoracolumbar	5.70 (4.59–7.12)	5.37 (3.92–6.81)*
**LV dimensional measure**			
IVSd (cm)	All SCI	1.0 (0.9–1.1)	1.0 (0.9–1.1)
	Cervical	1.0 (0.9–1.2)	0.9 (0.9–1.0)
	Thoracolumbar	0.9 (0.9–1.0)	1.0 (0.9–1.1)
LVIDd (cm)	All SCI	4.8 (4.5–5.1)	4.7 (4.4–5.0)
	Cervical	5.0 (4.6–5.3)	**4.9 (4.4–5.2)** ^†^
	Thoracolumbar	4.6 (3.9–5.1)	4.5 (3.9–5.0)
PWd (cm)	All SCI	0.9 (0.9–1.0)	1.0 (0.9–1.0)
	Cervical	1.0 (0.9–1.1)	1.0 (0.9–1.1)
	Thoracolumbar	0.9 (0.9–1.0)	1.0 (0.9–1.0)
IVSs (cm)	All SCI	1.5 (1.2–1.7)	1.3 (1.3–1.4)
	Cervical	1.6 (1.3–1.7)	1.3 (1.2–1.4)
	Thoracolumbar	1.3 (1.2–1.7)	1.3 (1.2–1.4)
LVIDs (cm)	All SCI	3.3 (3.0–3.5)	3.5 (3.1–3.5)
	Cervical	3.2 (2.9–3.7)	3.5 (3.1–3.7)
	Thoracolumbar	3.3 (2.7–3.6)	3.4 (3.0–3.5)
PWs (cm)	All SCI	1.5 (1.3–1.7)	1.5 (1.4–1.6)
	Cervical	1.6 (1.3–1.9)	1.5 (1.4–1.7)
	Thoracolumbar	1.4 (1.3–1.2)	1.5 (1.3–1.6)
RWT	All SCI	0.39 (0.37–0.45)	0.44 (0.38–0.46)
	Cervical	0.40 (0.35–0.46)	0.43 (0.38–0.46)
	Thoracolumbar	0.38 (0.37–0.46)	0.45 (0.37–0.49)
Estimated LV mass (g)	All SCI	170 (130–198)	171 (143–185)
	Cervical	188 (157–216)	180 (141–187)
	Thoracolumbar	145 (107–180)	169 (116–181)
Estimated LV mass index (g/m^2^)	All SCI	85 (57–97)	85 (65–90)
	Cervical	88 (72–107)	81 (68–91)
	Thoracolumbar	82 (48–91)	88 (55–90)

Values are median with lower and upper quartiles. Effects from Mann-Whitney U test performed for between-group comparisons. Between the 3- and 6-month time points for the SCI groups, a Wilcoxon signed-rank test for within-group comparisons. A, late diastolic mitral filling velocity; Q, cardiac output; d, diastolic; E, early diastolic mitral filling velocity; E', early myocardial relaxation tissue Doppler velocity; EDV, end-diastolic volume; ESV, end-systolic volume; IVS, interventricular septum; IVRT, isovolumetric relaxation time; LV, left-ventricle; LVID, left ventricular internal diameter; PW, posterior wall; RWT, relative wall thickness; s, systolic; S', systolic myocardial contractile tissue Doppler velocity; SCI, spinal cord injury; SV, stroke volume. Between-group comparison (i.e., thoracolumbar vs. cervical): *p < 0.05, **p < 0.01. Within-group comparison between 3- and 6- months: ^†^p < 0.05. All significant values are bolded at the 6-month time point.

There were no differences between the cervical SCI and thoracolumbar SCI groups for echocardiographic measures at 3- or 6-month time points, except for EF (*P* < 0.01), which was still within the normal range of 52–72% for males as per ASE guidelines (20). LV mechanics parameters can be found in Table 3. For LV systolic mechanics, apical rotation was higher in the thoracolumbar group at 6-months compared to 3-months, whereas radial strain rate at the mid-level was lower.

**Table 3:** Echocardiographic indices for LV mechanics between cervical and thoracolumbar SCI groups and within 3- and 6-month time points.

	**GROUP**	**Time** **since injury** **3 months**	**Time** **since injury** **6 months**
**Systolic peak**			
Basal rotation (°)	All SCI	−7.5 (−9.2 to −6.3)	−7.4 (−9.4 to −6.4)
	Cervical	−8.0 (−9.4 to −6.4)	−8.4 (−10.2 to −6.9)
	Thoracolumbar	−7.0 (−10.7 to −6.2)	−7.1 (−9.9 to −6.2)
Apical rotation (°)	All SCI	10.6 (9.4–11.5)	12.1 (9.2–13.9)
	Cervical	10.1 (6.8–10.6)	9.36 (7.47–11.40)
	Thoracolumbar	11.4 (10.4–14.3)	**13.3 (12.1–16.9)** ^*^
Twist (°)	All SCI	17.6 (16.3–21.5)	20.35 (17.49–24.1)
	Cervical	17.2 (15.6–18.2)	17.6 (14.9–21.2)
	Thoracolumbar	20.8 (16.8–23.7)	21.8 (20.4–24.3)
Torsion (°/cm)	All SCI	1.9 (1.7–2.5)	2.3 (1.8–2.5)
	Cervical	1.7 (1.6–1.9)	1.8 (1.4–2.3)
	Thoracolumbar	2.5 (1.7–2.5)	2.2 (2.3–2.6)
Longitudinal strain (%)	All SCI	−18 (−19 to −17)	−18 (−19 to −17)
	Cervical	−17 (−19 to −16)	−17 (−18 to −16)
	Thoracolumbar	−18 (−21 to −17)	−19 (−19 to −18)
Mid radial strain (%)	All SCI	41 (30–61)	43 (35–57)
	Cervical	37 (30–45)	43 (39–53)
	Thoracolumbar	61 (29–65)	35 (27–64)
Mid circumferential strain (%)	All SCI	−23 (−30 to −21)	−22 (−25 to −20)
	Cervical	−25 (−36 to −23)	−22 (−25 to −19)
	Thoracolumbar	−21 (−26 to −18)	−21 (−28 to −21)
Basal rotation rate (°/s)	All SCI	−75.3 (−95 to −64)	−74 (−91 to −50)
	Cervical	−82 (−126 to −70)	−78 (−107 to −47)
	Thoracolumbar	−64 (−84 to −62)	−69 (−86 to −55)
Apical rotation rate (°/s)	All SCI	104 (83–136)	104 (86–120)
	Cervical	102 (74–125)	104 (58–107)
	Thoracolumbar	107 (92–137)	117 (95–149)
Longitudinal Strain Rate (s^−1^)	All SCI	−1.09 (−1.19 to −0.96)	−1.01 (−1.05 to −0.94)
	Cervical	−1.07 (−1.32 to −0.94)	−1.00 (−1.03 to −0.90)
	Thoracolumbar	−1.11 (−1.15 to −0.96)	−1.04 (−1.11 to −0.91)
Mid circumferential strain rate (s^−1^)	All SCI	−1.29 (−1.91 to −1.18)	−1.22 (−1.49 to −1.08)
	Cervical	−1.19 (−1.97 to −1.18)	−1.17 (−1.35 to −0.96)
	Thoracolumbar	−1.29 (−1.79 to −1.19)	−1.39 (−1.71 to −1.04)
**Diastolic peak**			
Basal rotation rate (°/s)	All SCI	63 (57–82)	59 (45–81)
	Cervical	63 (60–74)	56 (46–83)
	Thoracolumbar	70 (41–111)	62 (44–83)
Apical rotation rate (°/s)	All SCI	−109 (−136 to −91)	−122 (−133 to −83)
	Cervical	−108 (−130 to −88)	−86 (−122 to −73)
	Thoracolumbar	−110 (−143 to −94)	−130 (−142 to −112)
Longitudinal strain rate (s^−1^)	All SCI	1.27 (1.14–1.43)	1.10 (1.02–1.37)
	Cervical	1.29 (1.23–1.34)	**1.07 (0.95–1.15)** ^†^
	Thoracolumbar	1.18 (0.97–1.66)	1.32 (1.02–1.54)
Mid radial strain rate (s^−1^)	All SCI	−3.60 (−4.59 to −2.19)	−3.15 (−3.95 to −2.45)
	Cervical	−3.20 (−4.29 to −2.14)	−2.83 (−4.05 to −2.46)
	Thoracolumbar	−4.01 (−5.27 to −1.88)	−3.47 (−4.16 to −2.07)
Mid circumferential strain rate (s^−1^)	All SCI	1.26 (1.04–1.59)	1.24 (1.13–1.69)
	Cervical	1.27 (0.99–1.91)	1.20 (0.88–1.70)
	Thoracolumbar	1.26 (0.92–1.49)	1.28 (1.13–1.85)

Values are median with lower and upper quartiles. Effects from Mann–Whitney U test performed for between–group comparisons. Between the 3- and 6-month time points for the spinal cord injury (SCI) groups, a Wilcoxon signed-rank test for within-group comparisons. Between-group comparison (i.e., thoracolumbar vs. cervical): ^*^p < 0.05. Within-group comparison between 3- and 6-months:^†^p < 0.05. All significant values are bolded at the 6-month time point.

## Discussion

To our knowledge, this is the first study to investigate the time-course of cardiac changes in humans with sub-acute SCI (i.e., serial echocardiography measurements performed within the first 6 months post injury). While a recent meta-analysis demonstrated that structural and functional changes are apparent for individuals with chronic SCI compared to non-injured individuals (1), these current findings suggest changes may occur during the sub-acute stage post cervical SCI. The novel aim of this study was to longitudinally track the same individuals in each group over a 3-month period in the sub-acute stage of injury. The reduced cardiac capacity for the cervical SCI group may limit the ability of these individuals to perform regular activities of daily living (16), consequently putting these individuals at a greater risk for developing chronic diseases (i.e., CVD) (26) and impacting aspects of health-related quality of life.

Reductions in LV structural and functional cardiac indices were found for individuals with cervical SCI between 3- and 6-months post-injury, whereas these changes were not apparent for individuals with thoracolumbar SCI. As the LTPA measures were highly variable but somewhat comparable amongst the cervical SCI and thoracolumbar SCI participants, the most likely explanation for the differences between groups can be attributed to the disrupted supraspinal regulation of sympathetic activity to the heart following cervical SCI (5). To support this, our group has previously published translational work suggesting the mechanism behind the reduction in cardiac function is underpinned by interrupted bulbospinal sympathetic control (27). Conversely, cardiac sympathetic control is preserved in those with thoracolumbar SCI. It has been demonstrated in individuals with chronic SCI that a higher NLI is likely to result in more devastating consequences for cardiac function (28). However, these longitudinal cardiac adaptations, assessed using echocardiography, have never previously been captured in the first 6 months following human SCI with the same individuals.

Despite reductions in LV structure and global function in the cervical SCI group, indices of LV systolic mechanics were similar over time. Perhaps this can be attributed to a mechanical compensation post-SCI to maintain systolic function in the cervical SCI group. The observation of preserved systolic mechanics and reduced SV has been previously reported (29), and is thought to be attributed to reduced afterload from the chronically hypotensive state of individuals with SCI, even with reductions in preload.

### Leisure Time Physical Activity

Despite recommended exercise guidelines to improve cardiometabolic health following SCI (3), 40 and 50% of the SCI participants at 3- and 6-months post-injury, respectively, self-reported 0 min of moderate-to-heavy intensity LTPA per week. This is in agreement with previous literature revealing that 50% of community-dwelling individuals with SCI do not perform any LTPA (30). While the LTPAQ-SCI does not include activities of daily living in its assessment, literature suggests that activities of daily living are not associated with cardiovascular fitness in this population (31) and are therefore unlikely to have an impact on the cardiac indices measured in this study. During in-patient rehabilitation in Canada, participants have self-reported performing > 60 min of vigorous-intensity physical activity per day (31). However, the same study indicated that only 5 min per day was spent performing physical activity > 40% heart rate reserve_._ Therefore, self-reported minutes of moderate-to-vigorous-intensity physical activity do not reflect actual time spent performing activities at higher intensities as measured objectively *via* a heart rate monitor. Therefore, In-keeping with other results, it could be implied that individuals with SCI do not reach an effective cardiovascular training threshold to improve neurologic, cardiovascular, or musculoskeletal health during inpatient therapy (31). This suggests further work to optimize rehabilitation strategies is warranted.

Previous literature suggests the cardiovascular deconditioning following SCI can be improved with exercise training (2). However, given the reduced upper-extremity motor scores (range 11–40), individuals with cervical SCI can struggle to perform volitional upper-body exercise of a sufficient intensity or volume to offset CVD risk. These reduced upper-extremity motor scores may reflect an overestimation of reported LTPA, though this suggestion is outside the scope of our study. Thus, individuals with cervical SCI may require alternative forms of exercise to prevent cardiac decline. Pre-clinical rodent models have shown improvements in LVIDd, EDV, and SV following passive hind-limb exercise (32), suggesting passive lower-limb manipulation or stimulation (33) may improve cardiac function (34).

We contend that disrupted sympathetic control to the heart and blood vessels after cervical SCI (5) is primarily responsible for the accelerated cardiac decline in these participants. Physical activity may mitigate this to some degree (35), but the effectiveness of volitional upper-body exercise, especially in individuals with cervical SCI during the sub-acute phase requires further research. Additional work is required to promote exercise in humans with SCI in the sub-acute setting and investigate the efficacy of different exercise intensities and modalities to improve or maintain cardiac health.

### Future Directions

Our novel findings, while preliminary and requiring verification with a larger cohort, reveal that significant cardiac changes occur in the sub-acute period following cervical SCI. It would be of interest to track these individuals into the chronic period of injury to observe whether these changes plateau or decline further. In addition to exercise, early pharmacological strategies may mitigate this cardiac decline, which potentially has implications for the risk of developing CVD. A recent pre-clinical study investigating the use of dobutamine in the acute setting to augment LV contractility, appears to preserve cardiac function in the chronic setting post-SCI (36). Furthermore, novel neuromodulation strategies, such as the application of epidural stimulation (37) to facilitate supraspinal control of the sympathetic nervous system could complement acute rehabilitation strategies. Although the cardiac indices measured in our participants are somewhat variable and within the normal range compared to clinical guidelines (20), the major strength of our study is the longitudinal design, which tracks the same participants during this novel sub-acute period.

### Study Limitations

Although our groups are comparable for demographic characteristics, the progression of each type of injury can only be truly compared by measuring indices prior to injury. This is an implausible study design; therefore, pre-clinical models would help to complement these findings. Furthermore, while NLI and severity of SCI were reported using validated methods, autonomic completeness of the injury was not measured. However, associations between sensorimotor and sympathetic impairments have previously been observed in non-athletic individuals with SCI (38). Consequently, it is not possible to state with certainty that these findings are due to disrupted supraspinal cardiac control. MH-LTPA variations also emphasize the potential limitations of using self-report questionnaires. Participants were taken from a sample of inpatients at a single, local rehabilitation center who had access to transportation for testing appointments following discharge, therefore potentially subjecting the study to a degree of sampling bias. These individuals were not highly trained athletes or deconditioned before injury, therefore our sample represents individuals from an urban developed country that have sustained a SCI. Future research should incorporate validated, research-grade multi-sensor devices (39) to precisely quantify physical activity levels and to ascertain how the modification of certain physical activity dimensions might alter cardiac indices in the sub-acute setting. TTE recommendations to scan participants in the left-lateral supine position to avoid foreshortening (20) may present a limitation, as reductions to preload in an upright position (1) are common in individuals with SCI and should be considered for future studies. Finally, the role of vasoactive medications on these cardiac findings would be of interest in future studies as the optimal dose of these pharmaceutical strategies are still being investigated, particularly in the sub-acute period following injury (40).

## Conclusion

Our data demonstrate a decline in several cardiac indices using TTE following cervical SCI, as early as 6-months post-injury. A lack of descending sympathetic control of the heart may be responsible for the rapid adaptive changes in cardiac indices. Worryingly, these changes occur over a relatively short time frame following cervical SCI. This highlights the need to investigate the effectiveness of different therapeutic strategies to mitigate the decline of cardiovascular function in this population during the sub-acute period.

## Data Availability Statement

The raw data supporting the conclusions of this article will be made available by the authors, without undue reservation.

## Ethics Statement

The studies involving human participants were reviewed and approved by University of British Columbia Clinical Research Ethics Board (H13-03072). The patients/participants provided their written informed consent to participate in this study.

## Author Contributions

MW and AK had full access to all the data in the study and take responsibility for the integrity of the data and the accuracy of the data analysis. SB, TN, MW, KC, and AK contributed to conception and experimental design. SB acquired the data. SB, TN, and MW analyzed the data. SB, TN, KC, CW, TT, MW, and AK interpreted the data. SB and TN drafted the manuscript. SB, MW, TN, KC, CW, TT, and AK contributed to editing the final version of the manuscript. AK is the principle investigator of the project. TN and MW co-supervised the project. All authors contributed to the article and approved the submitted version.

## Funding

This work was supported by a grant (Principal Investigator: AK) from the Craig H. Neilsen Foundation (Grant H13-03072), the Canadian Foundation of Innovation (CFI; 35869 - John R. Evans Leaders Fund), and the BC Knowledge Development Fund (BCKDF; 53869). SB was supported by The Robert H.N. Ho Scholarship, UBC President's Academic Excellence Award, and Peter McLardie Award (Sonography Canada). TN was supported by a Postdoctoral Research Trainee Award from the Michael Smith Foundation for Health Research (MSFHR) in partnership with the International Collaboration on Repair Discoveries, (Grant Number: 17767). KC was supported by a Craig H. Neilsen Foundation Postdoctoral Fellowship (Grant Number: 281863). MW was supported by a Postdoctoral Research Trainee Award from MSFHR in partnership with the Rick Hansen Foundation (Grant Number: 17110). AK holds the Endowed Chair in Rehabilitation Medicine.

## Conflict of Interest

The authors declare that the research was conducted in the absence of any commercial or financial relationships that could be construed as a potential conflict of interest.

## Publisher's Note

All claims expressed in this article are solely those of the authors and do not necessarily represent those of their affiliated organizations, or those of the publisher, the editors and the reviewers. Any product that may be evaluated in this article, or claim that may be made by its manufacturer, is not guaranteed or endorsed by the publisher.

## Supplementary Material

Supplementary Material 1 STROBE statement checklist.
